# mTOR inhibitors: A novel class of anti-cancer agents

**DOI:** 10.1186/1750-9378-7-1

**Published:** 2012-01-03

**Authors:** Haris Riaz, Talha Riaz, Syed A Hussain

**Affiliations:** 1 Dow University of Health Sciences, Karachi, Pakistan; 2 Intern, Civil Hospital Karachi, Pakistan; 3 Medical Student, Dow Medical College, Karachi, Pakistan

## 

Mammalian Target of Rapamycin (mTOR) is a serine/threonine protein kinase that acts as a master switch between anabolic and catabolic functions of the human body in pathways stimulated by insulin, growth factors and mitogen [1]. mTOR functions as a central controller of growth, proliferation, metabolism and angiogenesis, but its signaling is dysregulated in various human diseases especially certain cancers like renal cell carcinoma and breast cancer [2]. In cancer, mTOR is frequently hyperactivated which promotes cancer development and progression. In certain cancers, resistance to antineoplastic agents such as topoisomerase 1, topoisomerase 2 inhibitors and methotrexate can be overcome with a synergistic combination with mTOR inhibitors [3,4]. Furthermore, mTOR activates the degradation of cyclin dependent kinases such as CDK1 which increases synthesis of dihydrofolate reductases. By decreasing this enzyme, mTOR inhibitors like sirolimus and temsirolimus, promote tumour sensitivity to agents such as methotrexate [4].

Recent development has made cancer treatment move on from conventional cytotoxic drugs to agents that target specific proteins like mTOR called mTOR inhibitors. A very common mTOR inhibitor, rapamycin, is a bacterial product that inhibits mTOR by associating with its intracellular receptor [5]. [Currently, two mTOR inhibitors, temsirolimus and everolimuswhich are derivatives of rapamycin, temsirolimus(Torisel: Wyeth-Ayerst, Charlotte, NC, U.S.A.) and everolimus(Certican: Novartis Pharmaceuticals, St. Louis, MO, U.S.A.) ] are approved for the treatment of patients with advanced renal cell carcinoma (RCC) and mantle cell lymphoma, effectively translating this paradigm into the clinical setting [6].

mTOR inhibitors (like other drugs) have an adverse effect profile. Clinical trials have had mixed opinions regarding drug efficacy [7]. Examples of the neoplasias with promising results include pancreatic neuroendocrine tumors, follicular lymphoma, renal cell carcinoma and mantle cell lymphoma while the ones with negative results include glioblastoma multiforme and small cell carcinoma of lung. Although relatively safe, these drugs are associated with some unique adverse side effects, such as hyperlipidemia, hyperglycemia, and pneumonitis, which require monitoring and may require clinical intervention [6]. Clinical utility of mTOR inhibitors depends on appropriate selection of patients and type of cancer. Mutations in the mTOR pathway of cancer cells may result in resistance to mTOR inhibition and prevent any action of the mTOR inhibitors. Examples include mutations of FKBP-12 proteins, mammalian 14-3-3 proteins ATM (ataxia telangiectasia, mutated) cells, all responsible for growth of cancer cells.

A new wave of clinical trials has commenced using a second generation of mTORC1 and mTORC2 inhibitors. First generation of mTOR inhibitors like rapamycin, showed certain limitations by blocking only C1 isoform, inducing feedback activation of Akt and showing resistance to mTORC2 [8]. The newer agents can inhibit both mTORC1 and mTORC2 by targeting kinase domains as an effective means with a high degree of selectivity [9]. For example, Agent OSI-027 (OSI Pharmaceuticals, Melville, NY, U.S.A.) is currently in phase 1 of trial and being evaluated on patients with lymphoma or solid tumors [9]. XL765 (Exelixis, San Francisco, CA, U.S.A.) is also in phase 1 of clinical trial and being assessed in combination therapies [9].

In contrast to the older mTOR inhibitors like rapamycin which blocked only C1 isoform, the newer agents can inhibit both mTORC1 and 2 with high degree of selectivity [10]. Further clinical trials are necessitated to determine the therapeutic uses, predictive biomarkers and clinical efficacy for this novel class of anti-cancer agents.

## Authors' contributions

HR: Decided the topic to write about and made edits in resubmission. TR: Found the literature regarding the topic. SAH: Wrote the initial manuscript. All authors read and approved the final manuscript
